# A Low Dimensional Approach on Network Characterization

**DOI:** 10.1371/journal.pone.0109383

**Published:** 2014-10-16

**Authors:** Benjamin Y. S. Li, Choujun Zhan, Lam F. Yeung, King T. Ko, Genke Yang

**Affiliations:** 1 Department of Electronic Engineering, City University of Hong Kong, Hong Kong, Hong Kong; 2 Department of Electronic and Information Engineering, The Hong Kong Polytechnic University, Hong Kong, Hong Kong; 3 Department of Automation, Shanghai Jiao Tong University, Shanghai, China; Mathematical Institute, Hungary

## Abstract

In many applications, one may need to characterize a given network among a large set of base networks, and these networks are large in size and diverse in structure over the search space. In addition, the characterization algorithms are required to have low volatility and with a small circle of uncertainty. For large datasets, these algorithms are computationally intensive and inefficient. However, under the context of network mining, a major concern of some applications is speed. Hence, we are motivated to develop a fast characterization algorithm, which can be used to quickly construct a graph space for analysis purpose. Our approach is to transform a network characterization measure, commonly formulated based on similarity matrices, into simple vector form signatures. We shall show that the 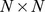 similarity matrix can be represented by a dyadic product of two *N*-dimensional signature vectors; thus the network alignment process, which is usually solved as an assignment problem, can be reduced into a simple alignment problem based on separate signature vectors.

## Introduction

In recent years, network mining has received a considerable amount of attentions. One important aspect of network mining is to measure the dissimilarities among networks since they can provide information to reproduce a graph space and allow analysis to be performed [1],[2]. Yet due to the complex nature of networks, this is considered to be a challenging task [1]–[4].

Although it is challenging, many algorithms have been developed to solve the network comparison problem. Umeyama formulated the problem into a combinatorial optimization problem and was solved via eigendecomposition [5], Singh et al. proposed a Page rank like similarity matrix IsoRank and employed it on the search of optimal assignment [6], Li et al. proposed an integer quadratic programming approach and was solved using an ellipsoid trust region method with interior point technique [7], etc. These methods mainly consider a one-to-one comparison and may not be efficient to handle large data set. Signature extraction is one effective way to treat such large volume of data. While representing the data with a signature vector, the comparison among complex objects can be reduced to comparison between signature vectors. In addition, for pairwise comparisons, the aforementioned optimization problem is no longer required to be solved in every pairs of networks. A typical type of signature vector is the motif count vector, which summarizes the network structures by the occurrence frequencies of specific subgraphs [8]–[12]. Although many effective algorithms are being designed for motif counting, the computational demand is still high and the computation complexity increases as more motifs are considered.

In this paper, an alternative approach with a balance between precision and computational efficiency is proposed. Eigenvector signature distance (EVSD) is a dissimilarity measure for large-scale pairwise network comparison based on signature vector extraction techniques. The basic idea of EVSD is to represent a network by a signature vector, which is the Perron-Frobenius (PF) vector of a network's adjacency matrix. In this paper we shall show that the network comparison and alignment problem can be reduced from a matrix alignment problem into a vector alignment problem. Consequently, this vector alignment problem can be solved by simple sorting operations. Hence the complexity is reduced from 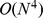 to 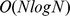.

The optimal distance EVSD can be further reconstructed into an agreement measure, eigenvector signature agreement (EVSA), which can be used to quantify the similarity between two networks. The distribution of EVSA has been studied through pairwise comparisons of artificially generated networks. Results have shown that EVSAs of networks with similar structure are notably higher than EVSAs of networks with dissimilar structures. In addition, comparison between EVSA and another state-of-art signature induced similarity measures, Graphlet Degree Distribution Agreement (GDDA), will be given in Section 3.2.2. The comparison results show that classifications based on EVSA have a relatively stable distribution, which can provide a more convincing and consistent inference.

## Methods

In this section we first formulate the network comparison problem, and then we show how the problem can be reduced and solved via the decomposition of Blondel's similarity matrix. Finally, based on the solution of this problem, we introduce Eigenvector Signature Distance and Eigenvector Signature Agreement to quantify networks' dissimilarity and similarity respectively

### 2.1 Preliminary

A graph 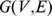 consists of the vertices set 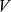 and the edges set 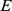. The edges set is a collection of all edges, each edge can be represented in the form of 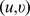, where 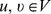. A network can be quantified by a number of statistical measures. For instance, degree of node 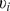, 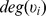, which is the total number of edges connected to node 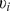. The average degree, 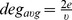, where 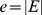 and 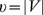 respectively. The graph density 
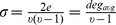
, is the ratio of the number of existing edges to the largest possible number of edges.

A graph 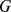 can also be represented by its adjacency matrix, 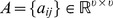, entry 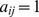 shows that there is an edge connected from node 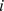 to node 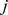, otherwise 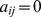. If the graph is undirected, the adjacency matrix will be symmetric. If the graph is connected, the adjacency matrix is irreducible.

### 2.2 Network Comparisons and Signature Vector

The network comparison problem can be considered as finding a graph distance metric *d*, which quantifies the difference between networks. There are many candidate measures that can be used [13]–[15]. Most of these measures need to deal with the problem caused by large amount of vertex mapping variations. For instance, if two networks of size 10 are being compared, then there exist 3628800 variations of vertex mapping. Computing the graph distance using the metric *d* on all the vertex mappings will be computationally demanding. Picking an arbitrary vertex mapping may yield a distance measure that is inappropriate for comparison.

An appropriate measure would be,
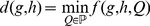
(1)where 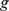 and 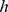 are graphs, 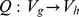 is a mapping that maps nodes in graph 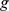 to 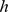, 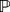 is the set of all permutation matrices. 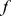 represents a metric that quantifies dissimilarity between 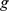 and 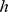 under the mapping 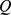. A practical formulation of (1) could be designed with the aid of a similarity matrix. Similarity matrix is a node based similarity measure which stores all node-to-node pairwise similarity information between two networks. With such matrix, the problem can be transformed into searching a suitable mapping of nodes between two networks, which at the same time maximize the sum of all node pair similarities. That is
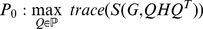
(2)where 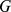 and 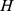 are the adjacency matrices of graph 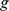 and 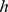 respectively. 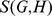 is the similarity matrix which 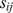 is the similarity between node 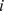 in network 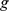 and node 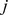 in network 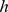.

Note that, there are more than one way to interpret similarity between two networks. In this paper we employed the similarity matrix proposed by Blondel et al. [16]. According to Blondel et al., the similarity matrix for graphs 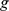 and 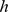 with adjacency matrices 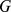 and 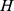 can be computed by the following iterative process.
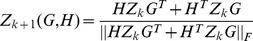
(3)where 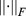 is the Frobenius norm. 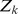 will finally converge to the similarity matrix.

While 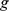 and 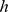 are undirected graphs, the iterative process can be simplified into
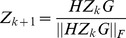
(4)


Thus 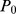 becomes
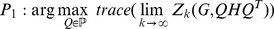
(5)


On the other hand, by multiplying 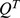 to both side of the iterative process, we have
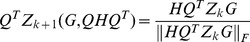
(6)


Note that 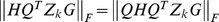 as Frobenius norm is unitarily invariant.

Let 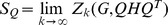 and 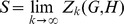, from (6) we have the following relationship
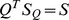
(7)


Thus 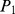 becomes
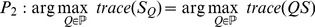
(8)



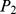
 is an 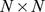 assignment problem which can be solved by the Hungarian method in 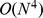 time. It is computational demanding for large 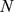. The efficiency can be dramatically reduced by the following decomposition on the 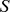 matrix.

According to the well know Von Mises iteration method [17], the following iterative processes converge and 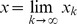, 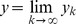 are the Perron-Frobenius (PF) vectors of 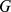 and 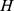 respectively.
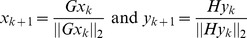
(9)


Let 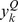 be defined by 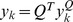. From (9) we have
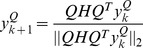
(10)


And we can see that 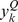 is the PF vector of 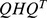. Note that 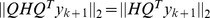 as Euclidean norm is unitarily invariant.

Combining (9) and (10) we have
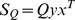
(11)


Thus 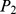 becomes
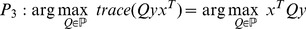
(12)


Since product of permutation matrices is still a permutation matrix, problem 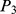 can be rewritten into the following form,
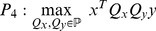
(13)and the problem becomes finding optimal permutation on 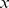 and 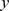 such that their inner product is maximized. According to the rearrangement inequality [18], the inner product is maximized when the two vectors are sorting in descending order. That is,
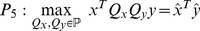
(14)where 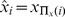 and 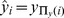, 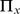 and 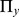 are the optimal mapping such that
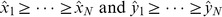
(15)


Here 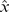 and 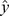 are considered as the eigenvector signatures (EVS) of the two networks. By combining equations (14) and (15), we can see that the original problem 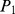 can be solved via a simple sorting algorithm with the aid of our proposed decomposition. Hence, once the eigenvector is computed, only 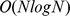 time is needed to complete the alignment. When comparing with the case without the decomposition, the computational time of 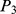 is reduced from 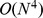 to 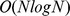 on the alignment process. In addition, in the case of pairwise comparison among 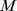 networks, as signatures are only required to be compute and sorted once, thus the time complexity of our proposed method is only 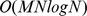. Yet without the decomposition, an assignment problem is required to be solved in each comparison, hence the time complexity of the entire task is 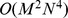.

### 2.3 Eigenvector Signature Distance and Eigenvector Signature Agreement

The optimal value of 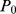 can be shown to be related to the Euclidean distance between EVSs of two networks. The relation can be summarized by the following property.

**Property 2.1** 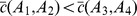 *iff* 
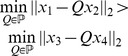
, *where* 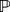 *is the set of permutation matrices*, 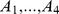 *are adjacency matrices and 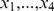 are their respective EVSs*.

*Proof of Property 2.1.* Suppose 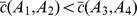, we have
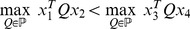
(16)


Hence
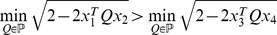
(17)


That is
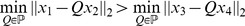
(18)


On the other hand, if 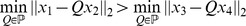,

we have
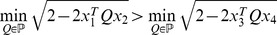
(19)which is equivalent to
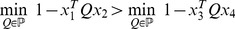
(20)


According to Perron-Frobenius Theorem, all entries of 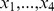 are nonnegatives, as 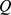 is a permutation matrix so entries of 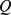 should also be nonnegatives. Thus,
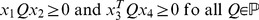
(21)


Therefore
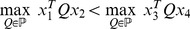
(22)


Hence,
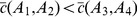
(23)


According to the above property, for topological similar networks, the Euclidean distance between their corresponding EVSs will be smaller and vice versa. This indicates that the Euclidean distance between EVSs can be considered as the measure of dissimilarity between networks. With this, a network measure can be defined by:
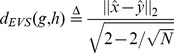
(24)where 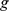 and 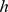 are graphs, 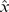 and 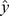 are their EVS respectively. The denominator 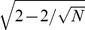 is to normalize the measure so that 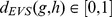. This choice of value can be explained by the following property.

**Property 2.2** *The farthest pair of vectors in the set* 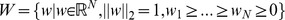 *are* 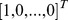 *and* 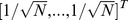.

*Proof of Property 2.2.* Let 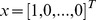, 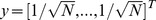 and 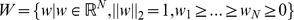.

We show 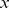 and 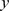 are the farthest pair of vectors in the set 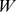 by contradiction.

Suppose 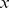 is not the farthest vector to 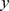 in the set 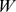. There exists a vector 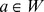 where
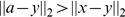
(25)

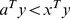
(26)

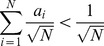
(27)

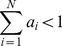
(28)


Since 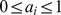


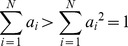
(29)which contradicts 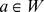.

Suppose 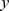 is not the farthest vector to 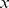 in the set 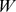. There exist a vector 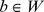 where
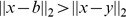
(30)

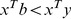
(31)

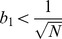
(32)


Since 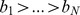


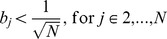
(33)


Thus
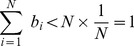
(34)which contradicts 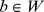.

Combine the above proofs, and the fact that 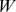 is a closed and connected subset of a unit sphere, it can be concluded that 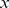 and 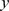 are the farthest vector pair in 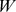.

In property 2.2, the set 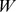 is the set of EVS, hence 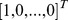 and 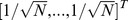 are the farthest pair of EVS and the maximal value of 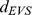 is 
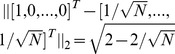
.

As 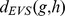 measures the difference between networks, alternatively, an agreement measure can be defined as a complement of EVSD,
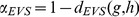
(35)


Eigenvector Signature Agreement (EVSA) is the network similarity measure induced by the EVS. It represents a measure of similarity between two networks; the higher the value, the more similar the networks. It is a normalized measure and lies within the range [0, 1]. An illustrative example of EVSA computation can be found in File S1.

## Results and Discussions

To illustrate the effectiveness of the proposed EVSA similarity score, standard test networks were used and will be given in Section 3.1. Then in Section 3.2, an application of EVSA is demonstrated with the analysis of protein-protein interaction (PPI) networks for a family of herpesvirus.

### 3.1 Control Test on Standard Network Models

In this section, a test based on artificially generated networks is conducted to illustrate the use of EVSA. The network models will be given in Section 3.1.1 and the results of the test can be found in Section 3.1.2. These models are chosen to conduct the algorithm test as their structure and properties are well known.

### 3.1.1 Network models

Four network models were chosen for testing: the Erdős Rényi random graph (ER), Barabási Albert model (BA), Geometric random graph (GEO), and Stickiness model (STICKY).

**Erdős Rényi random graph (ER)** A network is generated randomly without considering any geometric or probability distribution constraints. It starts with 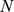 isolated nodes and 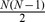 candidate edges. Candidate edges are all possible vertex pairs for edge being attached to which can be defined as the set 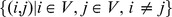. For each candidate edge, there is a constant probability 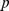 for an edge to be attached [19].

**Barabási Albert model (BA)** A scale-free network is generated through the preferential attachment scheme. Unlike the ER model, the probability of edges attachment in this model are not constant. It is directly proportional to the degree of nodes. Thus the resulting networks will reflect “the rich get richer” phenomenon. The degree distribution of a scale free network follows a power law. That is 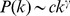, where 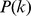 is the population of nodes having degree 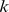, 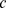 and 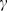 are constants [20].

**Geometric random graph (GEO)** A networks is generated randomly by the following procedures. Initially, nodes are randomly distributed in an *N*-dimensional Euclidean space. For any node pairs having geometric distance smaller than the threshold radius *r*, a link will be attached among them. In this paper the three-dimensional case is considered [21].

**Stickiness model (STICKY)** It is a network model designed for PPI networks. By providing the degree sequence of a network, the Stickiness model can be used to generate a set of networks having the same degree sequence. There are two main assumptions, i) the higher degree nodes have more reaction domain, i.e. these nodes can interact more frequently, ii) a stickiness index is defined; where a node pair both have a higher stickiness index, they are more willing to interact with each other. The stickiness index of nodes helps to control the expected degree sequence of the generated network [22]. The Stickiness model is designed to mimic a network based on the degree sequence, which is only used in the test among standard models; and in the study of herpesvirus PPI networks (Section 3.2), but not in the performance analysis (Section 3.2.2).

#### 3.1.2 Control Test Results

The control test was performed by first generating a reference network from each model, parameters were adjusted such that the size and average degree were 500 and approximately 10 respectively. Then perturbed the reference network by different ways: (a) randomly attaching 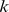 edges on the reference network, where 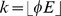, 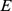 is the total number of edges in the reference network and 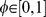 is an adjustable parameter for testing purpose (see Table 1); (b) randomly selecting 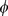 of the nodes in the reference network, replacing the interconnection of the selected nodes by a ER network; (c), (d) and (e) are similar to (b), but the injected network are GEO3D type, BA type and STICKY type respectively. EVSA between the reference networks and perturbed networks were then computed. The control test was repeated 50 times for each case and the mean EVSA were summarized in Table 1.

**Table 1:** This table summarized the EVSA values obtained from a series of control tests.

Type of Reference Network	*φ*	(a)	(b)	(c)	(d)	(e)
ER	0	1	1	1	1	1
	0.1	0.9814	0.9937	0.9935	0.9932	0.9934
	0.2	0.9716	0.9856	0.9869	0.9865	0.9873
	0.3	0.9607	0.9769	0.9778	0.9779	0.9770
	0.4	0.9513	0.9659	0.9683	0.9674	0.9669
	0.5	0.9440	0.9598	0.9597	0.9609	0.9582
GEO3D	0	1	1	1	1	1
	0.1	0.8061	0.9873	0.9683	0.9804	0.9693
	0.2	0.6914	0.9720	0.9410	0.9458	0.9460
	0.3	0.6213	0.9533	0.8760	0.9129	0.9156
	0.4	0.5705	0.9325	0.8477	0.8367	0.8841
	0.5	0.5323	0.8926	0.7994	0.8123	0.8262
	0	1	1	1	1	1
BA	0.1	0.9786	0.9898	0.9892	0.9900	0.9888
	0.2	0.9607	0.9735	0.9785	0.9732	0.9751
	0.3	0.9431	0.9616	0.9603	0.9520	0.9548
	0.4	0.9258	0.9466	0.9412	0.9279	0.9340
	0.5	0.9087	0.9304	0.9142	0.9120	0.9146

The first column indicates the network class of the reference network, the second column indicates the *φ* values in the control tests. Column (a) to (e) are the mean EVSA value obtained from 50 times of the corresponding test. (a) is the random edge attachment test, (b) is the ER injection test, (c) is the GEO3D injection test, (d) is the BA injection test and (e) is the STICKY injection test.

While the reference network was being perturbed by random attachment, most of the topology remained the same, thus in most of the cases the mean EVSA values were high. Yet in the case of GEO3D, the topology of reference network follows a geometric constrains, random attachment of edges violated this constrain and caused a large difference in topology. According to the results, randomly attaching 50% extra edges caused the mean EVSA score changed from 1 to 0.5323. On the other hand, in tests (b) to (e), the EVSA scores were found to be close among different types of injection with the same reference network and 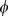 value. For instance, injecting a GEO3D network and BA network on a ER network with 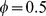 caused their mean EVSA values dropped from 1 to 0.9597 and 0.9609 respectively. This indicated that even the interconnection between part of the nodes were replaced by various types of network, the similarity between the original network and the perturbed network can still be reflected by their high EVSA.

### 3.2 Analysis on Protein-Protein Interaction Networks

In this section, five herpesviral PPI networks are analyzed using EVSA. The employed data set is described in Section 3.2.1, and the results can be found in Section 3.2.2.

#### 3.2.1 Protein-Protein Interaction Networks

The PPI network is a network that consists of proteins and their interactions within a single organism, for example baker's yeast and human. In the PPI network a protein is represented in the form of nodes. For every pair of proteins having interaction, a link will be attached in between the corresponding nodes. Here, five herpesvirus PPI networks were chosen for the evaluation, namely Epstein-Barr virus (EBV), herpes simplex virus (HSV), Kaposi's sarcoma-associated herpesvirus (KSHV), murine cytomegalovirus (mCMV) and varicella-zoster virus (VZV). These five herpesviral PPI networks were collected by Peter Uetz et al. [23],[24].

The following is an illustrative analysis on several PPI networks. The summary statistics of yeast and human PPI networks including graph density, are shown in Table 2. This is obtained from a study on GDDA [25]. According to that study, when the graph density is low, GDDA will suffer from a volatility issue.

**Table 2:** Summary Statistics of Yeast and Human PPI Network obtained from Rito et al.

Name	# Nodes	# Edges	Graph Density	Average Degree	Experiment Type	Organism	Reference
YIC	796	841	0.0027	2.11	Yeast two-hybrid	S. Cerevisiae	Ito et al. (2000)
YHS	988	2455	0.0050	4.97	TAP-MS	S. Cerevisiae	Von Mering et al. (2002)
HSHS	1705	3186	0.0022	3.74	Yeast two-hybrid	Homo Sapiens	Stelzl et al. (2005)
BG MS	1923	3866	0.0021	4.02	Affinity Capture-MS	Homo Sapiens	BIOGRID (filtered)

Here a similar analysis is conducted on five herpesvirus PPI networks: Epstein Barr virus (EBV), Herpes simplex virus (HSV), Kaposi's sarcoma-associated herpesvirus (KSHV), murine cytomegalovirus (mCMV) and varicella-zoster virus (VZV). The summary statistics can be found in Table 3. In the herpesvirus case, the graph density is relatively higher (0.06 to 0.12) than the yeast-human case (0.0007 to 0.005), which indicates that the low graph density property is not consistence in all PPI networks. This inconsistency may be caused by the definition of graph density.
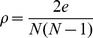
(36)


**Table 3:** Summary Statistics of HSV, VZV, KSPV, EBV, and mCMV PPI Network.

Name	# Nodes	# Edges	Graph Density	Average Degree	Experiment Type	Organism	Reference
HSV	48	100	0.0886	4.167	Yeast two-hybrid	Herpes simplex virus	Fossum et al. (2009)
VZV	55	159	0.1070	5.782	Yeast two-hybrid	Varicella zoster vrisu	Uetz et al. (2006)
KSPV	50	115	0.0938	4.6	Yeast two-hybrid	Kaposis sarcoma-associated herpesvirus	Uetz et al. (2006)
EBV	60	208	0.1175	6.933	Yeast two-hybrid	Epstein - Barr Virus	Fossum et al. (2009)
mCMV	111	393	0.0643	7.081	Yeast two-hybrid	Murine cytomegalovirus	Fossum et al. (2009)

Graph density is sensitive to network size since 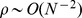, thus there exists an inconsistency between a small PPI network (e.g. herpesvirus) and a large PPI network (e.g. yeast). In this paper, instead of graph density, the average degree is being considered. The average degree is defined as
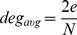
(37)


As 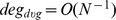, which is less sensitive to network size and hence is a more suitable candidate of changing variable in the performance evaluation for network similarity measures. This observation can be supported by the summary statistic as shown in Table 2 and 3; where average degree in yeast and human PPI network is around 2 to 5, in herpesvirus network it is about 4 to 7. It shows that average degree of node is more consistent than graph density among these three kinds of PPI networks. Note that by adjusting the average degree and network size, different ranges of graph density can also be covered.

#### 3.2.2 Results on Protein-Protein Interaction Networks

The procedure can be summarized as follows: first pick one of the herpesvirus PPI networks and compute its total number of edges, for each of the four models (ER, BA, GEO3D, STICKY), generate 50 candidate networks where the parameters are adjusted so that they have approximately the same number of edges as the herpesvirus networks. Then the EVSA score between the selected herpesvirus network and each of its candidate networks are computed, the EVSA scores are then averaged for each model. This average EVSA score is considered as the similarity score between the query network (e.g. EBV) and the testing model (e.g. ER). The process is repeated until all herpesvirus networks have been tested.

**Figure 1:**
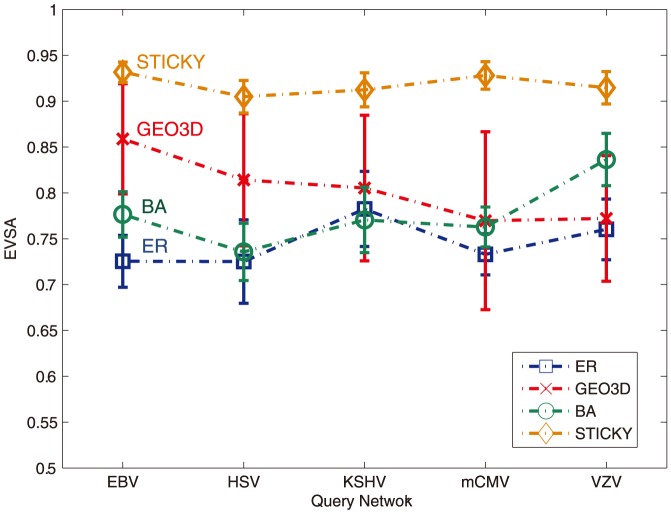
EVSA values of pairwise comparisons between PPI networks of EBV, HSV, KSHV, mCMV and VZV and network generated from ER,GEO3D,BA,STICKY models. The EVSA scores are averaged over 50 simulations and the error bar represents one standard deviation of the EVSA score.

Figure 1 shows the EVSA score of EBV, HSV, KSHV, mCMV and VZV matching with ER, GEO3D, SF and STICKY respectively. The EVSA scores between the STICKY model and the five herpesvirus networks are clearly higher than the other models. ER, GEO3D and BA have average EVSA scores around 0.8 and STICKY has average EVSA scores around 0.9. Since the STICKY model is distinct from the other three models in the matching test of herpesvirus PPI networks; we can observe that considering only ER, GEO3D, BA and STICKY models as candidates, STICKY is the closest model with respect to the herpesvirus family. This can also be interpreted that given only the degree sequence as information, one can reconstruct the PPI network of herpesvirus with similar topology using the stickiness model.

A similar analysis using GDDA was also conducted on the same five herpesviral PPI networks and yielded the same conclusion, STICKY is the best-fitting model of herpesvirus [26]. However, compared to the GDDA approach, the EVSA score is relatively stable among five herpesvirus PPI networks and the separation of EVSA scores between the STICKY model and the other network models is more apparent. This demonstrates that our results reinforce the observation of Kuchaiev et al. In Section 3.2.2, we will show that EVSA has higher stability and better classification performance.

## Performance Evaluation

Finally, the performance of network classification using EVSA is given and compared with another signature based network classification techniques: GDDA. In Section 4.1, the design of the testing is introduced. Volatility and classification performance of these two similarity score systems are compared in Sections 4.2 and 4.3 respectively.

### 4.1 Designs of Experiment

In order to illustrate the performance of different similarity score systems, models introduced in Section 3.1.1 was employed to generate networks for testing purposes. The experiment was conducted as follows.

Given two network models, for example ER model and BA model: in a ER-BA comparison, networks randomly generated from ER model using parameter set 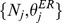 was considered as query network (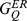), where 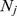 is the network size and 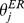 is the rest of ER model parameters. Similarly, 50 candidate networks 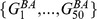 with same size 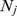 was randomly generated from BA model. The parameters 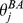 were adjusted such that the resulting candidate networks had approximately the same edge number to the one in 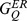.

The GDDA scores and EVSA scores between the query network and each of the candidate networks (i.e. 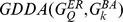 and 
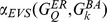
 for 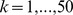) can then be computed. The process was repeated for several parameter sets 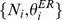, until all the interested 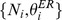 were considered. In this paper, the interested networks were limited to those with size 50, 100, 500, 1000, 2000 nodes and average degree 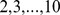. According to Section 3.2.1, these values cover most of the PPI networks of human, yeast and herpesviruses.

Comparisons between models can be separated into two classes; the match and the mismatch set comparisons. Match set comparisons are comparisons between networks generated from the same model, for instance, ER-ER comparison. Mismatch set comparisons are comparisons of networks generated across different models, for instance, ER-BA comparison. The score distribution of these two classes of comparison were used to illustrate the classification performance in Section 4.3.

### 4.2 Volatility of EVSA and GDDA

The stability of similarity score is an important issue as it directly affects the confidence of classification and the reliability of the scores. It can be measured as the standard deviation of a score among similar comparisons. An ideal similarity score should be non-volatile. Here our proposed similarity score EVSA is compared with GDDA in terms of volatility. The EVSA scores were computed using the algorithm mentioned in previous sections. GDDA scores were computed using GraphCrunch 2.

Table 4 shows the standard deviation of EVSA scores and GDDA scores in ER-ER, BA-BA and GEO3D-GEO3D comparisons with different average degrees respectively. According to Table 4, in most of the cases EVSA has a lower deviation as compare to GDDA. However, exceptions are found in low density cases and these exceptions are caused by the volatility of the model itself. In a recent study of network comparisons [27], topologies of low density or low average degree graphs are considered as highly volatile. A sensitive similarity measures could reflect this fact in terms of high deviation. This explained the relatively higher deviation of EVSA on lower average degree networks. For instance, in ER-ER comparison among networks with an average degree of 2, the standard deviation of EVSA is 0.0703 which is higher than that of GDDA (0.0565). On the other hand, in a higher average degree graph, the EVSA yields a relatively lower deviation which reflects the stability of the measure. For instance, in BA-BA comparison among networks with an average degree of 10, the standard deviation of EVSA is 0.0326 which is much lower than GDDA's (0.0737). Thus from this experiment, the higher sensitivity and robustness of EVSA is demonstrated.

**Table 4:** Standard deviation of EVSA, GDDA in same model comparison over various average degrees.

	Standard Deviation					
	ER-ER		BA-BA		GEO3D-GEO3D	
Average Degree	EVSA	GDDA	EVSA	GDDA	EVSA	GDDA
2	0.0703	**0.0565**	0.0688	**0.0649**	**0.0309**	0.0460
3	**0.0481**	0.0726	**0.0608**	0.0731	**0.0218**	0.0332
4	**0.0266**	0.0467	0.0563	**0.0539**	**0.0362**	0.0502
5	0.0369	**0.0334**	0.0521	**0.0518**	**0.0318**	0.0332
6	**0.0282**	0.0485	**0.0523**	0.0567	**0.0182**	0.0428
7	**0.0206**	0.0526	**0.0392**	0.0499	**0.0288**	0.0766
8	**0.0181**	0.0484	**0.0332**	0.0605	**0.0362**	0.0464
9	**0.0190**	0.0505	**0.0501**	0.0671	**0.0218**	0.0401
10	**0.0173**	0.0558	**0.0326**	0.0737	**0.0309**	0.0618

### 4.3 Classification Performance of EVSA and GDDA

Besides the volatility, the classification power of EVSA and GDDA is also an interesting aspect. The following is a test designed to reflect the classification power of a score. The basic idea of this test is to compare the distribution of scores in two cases, (i) networks generated from the same model and (ii) networks generated from different models.

In this experiment, networks were generated using ER, BA and GEO3D models. At the beginning of the test, a set of query networks was generated using different parameters through different models. For each of the networks in the query set, a number of candidate networks were generated using all three models. The parameters of the network model were chosen to generate networks with the same vertex number and approximately the same edge number. So the networks have approximately equal average node degree and graph density. With this set of candidate networks a set of GDDA and EVSA scores can be computed between each query network and their candidate networks. Here the GDDA scores were computed using the network analysis application GraphCrunch 2 [26].

The GDDA and EVSA scores were further classified into agreement scores of matching and mismatching sets respectively. Matching and mismatching agreements are the set of agreement scores where query networks and candidate networks are generated from the same and different model types respectively.

**Figure 2:**
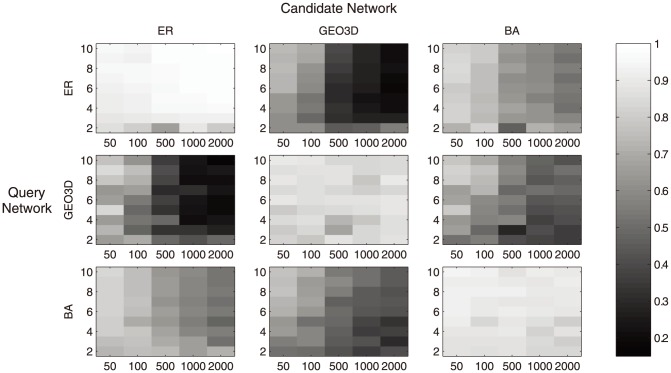
Heat map of average EVSA score in various comparisons. Each subplot represents the heat map of average EVSA score of a single type comparison. For instance, the bottom left subplot is the comparison of Scale-free (SF) network versus Erdos Renyi (ER) network, SF network is the Query Network and ER network is the candidate network. In each subplot, the y-axis represents the average degree, x-axis represents the network size (vertex number) the grey level represents the average EVSA score.

**Figure 3:**
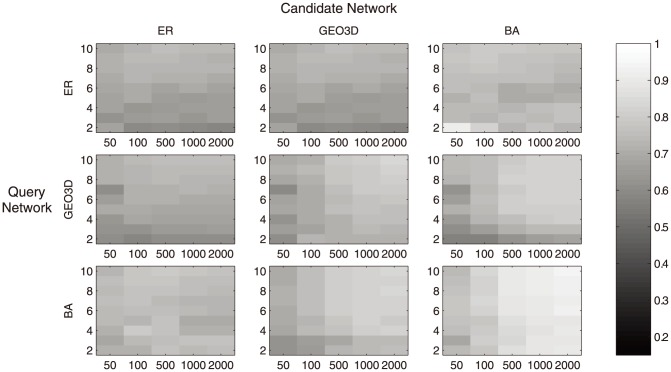
Heat map of average GDDA score in various comparisons. Each subplot represents the heat map of average GDDA score of a single type comparison. For instance, the bottom left subplot is the comparison of Scale-free (SF) network versus Erdos Renyi (ER) network, SF network is the Query Network and ER network is the candidate network. In each subplot, the y-axis represents the average degree, x-axis represents the network size (vertex number) the grey level represents the average.

The analysis results are summarized in Figures 2 and 3 in the form of a grey scale heat map. It shows the average GDDA and average EVSA in different parameter settings. The x-axis of the subplot represents the network size/vertex number; the y-axis represents the average degree of nodes. According to Figure 3, for GDDA, the difference between match-model comparisons (ER-ER, BA-BA, GEO3D-GEO3D) and mismatch-model comparisons (ER-BA, ER-GEO3D, BA-ER, etc) is not significant. While in the case of EVSA (Figure 2), notable difference is observed.

This reflects that the average EVSA scores among networks generated from the same model and those from different models have notable differences. It indicates that EVSA score can provide a clearer difference between the matching cases and the mismatching cases, or that the fuzziness and the mentioned difficulty on classification can be reduced.

**Figure 4:**
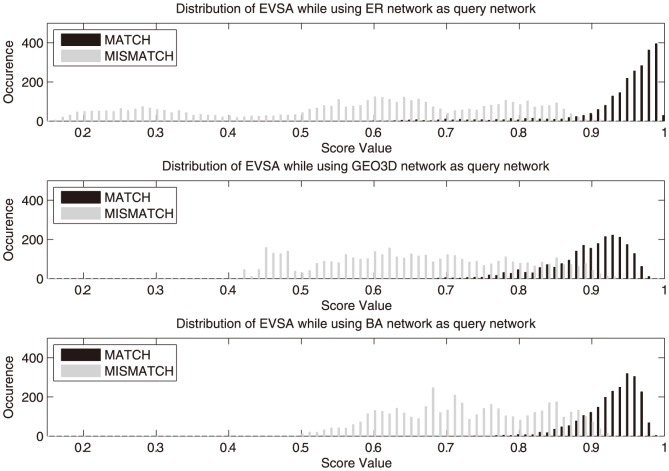
Sampled population of EVSA score. From top to bottom are a) ER network, b) GEO3D network, c) BA network as the query network used respectively.

**Figure 5:**
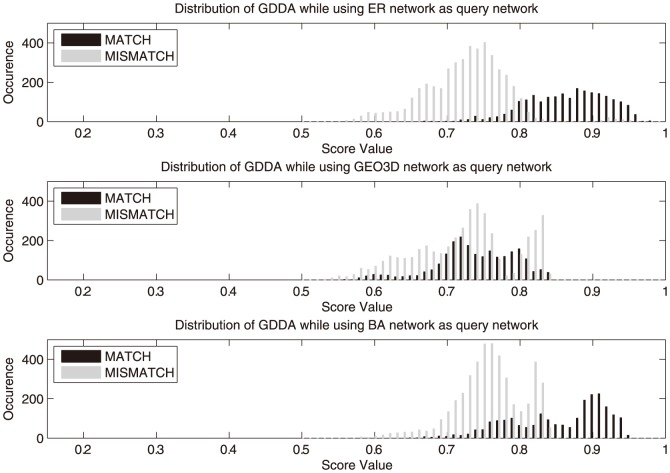
Sampled population of GDDA score. From top to bottom are a) ER network, b) GEO3D network, c) BA network as the query network used respectively.

To have a deeper understanding of the classification power of GDDA and EVSA, all the scores were being sampled and analyzed in terms of their distributions. The sampled data are illustrated in Figure 4 and 5 in histogram form. In EVSA, two sets of distributions can be found to be more divergent, which indicates that it provides a higher confidence for classification. In the GDDA case, the score distribution of matching models and mismatching models highly overlap when using GEO3D and BA networks as the query models. These overlapping regions are the “twilight zone” of the classification. The existence of such a region reduces the classification confidence and introduces fuzziness of classification.

To compare the divergence of matching and mismatching cases in GDDA and EVSA, the Jaccard distance is employed [28]. The Jaccard distance quantifies the divergence between two sets of distributions within a range from 0 to 1. A higher Jaccard distance indicates that the two distributions are more divergent and vice versa. Definition of Jaccard distance can be found in File S1.

Table 5 summarizes the Jaccard distance between matching and mismatching sets of GDDA and EVSA in three cases respectively: ER, GEO3D and BA type query networks. In GDDA, using ER network as the query network can yield a good Jaccard distance in classification (0.9149). While in cases using GEO3D and BA network as the query network, the classification power varies over a large range in terms of Jaccard distance (0.3060 and 0.7477). A good performance in a single case but less desire in other cases may lead to fuzzy inference results in classifying practical networks. In EVSA, all three types of query networks can yield a higher and more stable Jaccard distance around 0.9.

**Table 5:** The Jaccard Distance of GDDA and EVSA between matching and mismatching network sets.

	Jaccard Distance	
Query Network	EVSA	GDDA
ER	0.9942	0.9149
GEO3D	0.9149	0.3060
BA	0.9455	0.7477

On the other hand we also evaluated the classification performance of GDDA and EVSA via applying them on a support vector machine (SVM) classifier as kernel values [29], [30]. The SVM model classifies whether a network belongs or not to a specific class. The performances of the models are evaluated via a 5-fold cross validation and the results are summarized in Table 6. According to the results, model using EVSA as kernel value is relatively better that the one using GDDA in terms of accuracy, especially while classifying BA networks.

**Table 6:** The accuracy from 5-fold cross validation of SVM using EVSA and GDDA as kernel.

		Accuracy	
Class 1	Class 2	EVSA kernel	GDDA kernel
ER	Not ER	**81.48%**	77.78%
GEO3D	Not GEO3D	**92.59%**	81.48%
BA	Not BA	**96.30%**	66.67%

## Conclusions

In this paper the Eigenvector Signature is proposed. With this signature, the original matrix alignment problem in network characterization can be transformed into a vector alignment problem. Consequently the computational complexity can be drastically reduced from 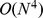 to 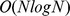. In addition, an agreement measure - Eigenvector Signature Agreement (EVSA) is designed to quantify the similarity between networks.

Experimental results have shown that EVSA has a stable classification behaviour among various settings of different network models, including ER random graph, BA scale free network and geometric 3D random network. An application of EVSA on classifying herpesvirus PPI networks had also been conducted. The results are consistent with the previous studies, with much faster speed. Furthermore, performance analysis between EVSA and another signature based graph similarity measure GDDA had also been conducted. Results show that EVSA can achieve higher stability and better classification performance. Moreover, since EVSA quantifies network similarity, it can also be considered as a graph kernel. Hence kernel-based learning algorithms can also be applied.

## Supporting Information

File S1 **Supporting information.** Definition S1: Jaccard Index and Jaccard Distance. Computation S1: Example of EVSA Computation.
(PDF)
