# Supplementary material for: A Low Dimensional Approach on Network Characterization
Source: PLoS One. 2014 Oct 16;9(10):e109383. doi: 10.1371/journal.pone.0109383 (PMC4199607; doi:10.1371/journal.pone.0109383)
Supplement: File S1 — Supporting information. Definition S1: Jaccard Index and Jaccard Distance. Computation S1: Example of EVSA Computation. (PDF) [file pone.0109383.s001.pdf]

Supplementary material: A Low Dimensional Approach on Network Characterization

Definition S1: Jaccard Index and Jaccard Distance

To illustrate the performance of GDDA and EVSA, the Jaccard Index was employed to quantify the divergence of scores between match and mismatch sets. Jaccard index is a statistic used to measure the similarity between sampled sets. It is defined as the ratio of size of intersection of the sample sets to the size of union of the sample sets, that is:

$$J(A,B) = \frac{|A \cap B|}{|A \cup B|} \tag{1}$$

To compute the Jaccard index of GDDA score and EVSA score, these two similarity scores are sampled into d bins. Let P_i^Z be the probability of a random sample taken from the set Z which belongs to bin i.

The Jaccard index can be rewritten into the following form [1].

$$S_{JAC} = \frac{\sum_{i=1}^{d} {P_i}^{Mat} {P_i}^{Mis}}{\sum_{i=1}^{d} \left({P_i}^{Mat}\right)^2 + \sum_{i=1}^{d} \left({P_i}^{Mis}\right)^2 - \sum_{i=1}^{d} {P_i}^{Mat} {P_i}^{Mis}} \tag{2}$$

where Mat is the set of score from match model comparison, Mis is the set of score from mismatch model comparison.

And the Jaccard distance can be defined as,

$$D_{JAC} = 1 - S_{JAC} \tag{3}$$

Jaccard index and Jaccard distance both lay between 0 and 1. In this case, the Jaccard index indicates how the distribution of matching score and mismatching score are overlapped. The overlapping region of scores is the grey area of the classification method, the larger the overlapping region, the higher the uncertainty while using the method to do classification.

Computation S1: Example of EVSA Computation

Given two graphs G_1 and G_2 with corresponding adjacency matrices A_1 and A_2.

$$A_1 = \begin{bmatrix} 0 & 1 & 0 & 0 & 0 \\ 1 & 0 & 1 & 0 & 0 \\ 0 & 1 & 0 & 1 & 0 \\ 0 & 0 & 1 & 0 & 1 \\ 0 & 0 & 0 & 1 & 0 \end{bmatrix} \text{ and } A_2 = \begin{bmatrix} 0 & 1 & 0 & 1 & 1 \\ 1 & 0 & 1 & 0 & 0 \\ 0 & 1 & 0 & 0 & 0 \\ 1 & 0 & 0 & 0 & 0 \\ 1 & 0 & 0 & 0 & 0 \end{bmatrix} \tag{4}$$

The eigenvector signature agreement (EVSA) between the two graphs can be computed as follows.

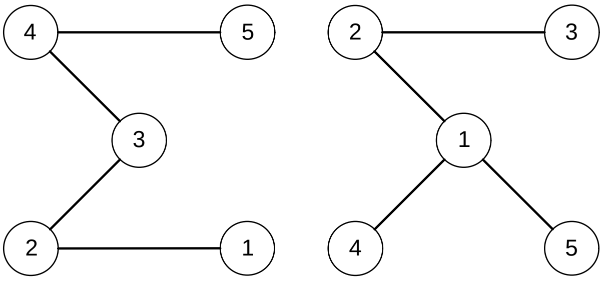

Figure 1: G_1(Left) and G_2(Right)

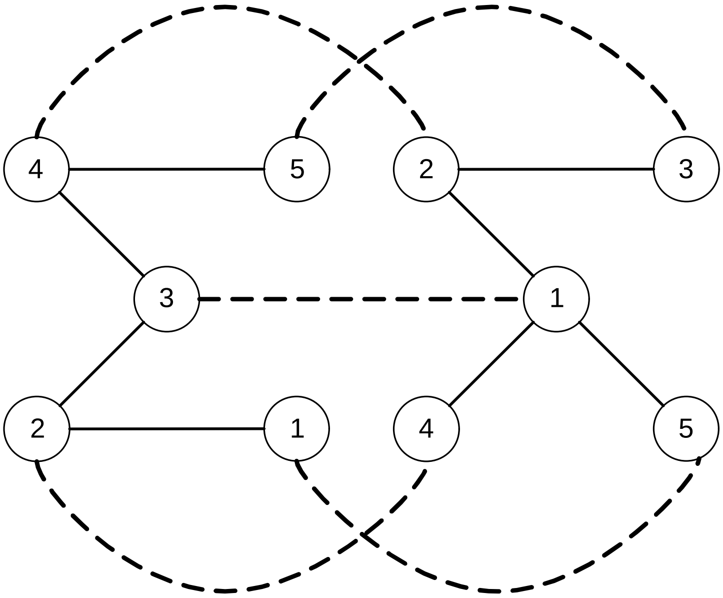

Figure 2: Visualization of the mapping Q^*

1. Find the normalized eigenvector with largest eigenvalue of A_1 and A_2.

$$x_1 = \begin{bmatrix} 0.2887 \\ 0.5000 \\ 0.5774 \\ 0.5000 \\ 0.2887 \end{bmatrix} x_2 = \begin{bmatrix} 0.6533 \\ 0.5000 \\ 0.2706 \\ 0.3536 \\ 0.3536 \end{bmatrix} \tag{5}$$

2. Sort in descending order

$$x^*{}_1 = Q_1 x_1 = \begin{bmatrix} 0 & 0 & 1 & 0 & 0 \\ 0 & 0 & 0 & 1 & 0 \\ 0 & 1 & 0 & 0 & 0 \\ 1 & 0 & 0 & 0 & 0 \\ 0 & 0 & 0 & 0 & 1 \end{bmatrix} \begin{bmatrix} 0.2887 \\ 0.5000 \\ 0.5774 \\ 0.5000 \\ 0.2887 \end{bmatrix} = \begin{bmatrix} 0.5774 \\ 0.5000 \\ 0.5000 \\ 0.2887 \\ 0.2887 \end{bmatrix} \tag{6}$$

$$x^*{}_2 = Q_2 x_2 = \begin{bmatrix} 1 & 0 & 0 & 0 & 0 \\ 0 & 1 & 0 & 0 & 0 \\ 0 & 0 & 0 & 1 & 0 \\ 0 & 0 & 0 & 0 & 1 \\ 0 & 0 & 1 & 0 & 0 \end{bmatrix} \begin{bmatrix} 0.6533 \\ 0.5000 \\ 0.2706 \\ 0.3536 \\ 0.3536 \end{bmatrix} = \begin{bmatrix} 0.6533 \\ 0.5000 \\ 0.3536 \\ 0.3536 \\ 0.2706 \end{bmatrix} \tag{7}$$

3. Eigenvector signature distance (EVSD) is the Euclidean Distance between x_1^* and x_2^*

$$||x_1^* - x_2^*||_2 = 0.1781 \tag{8}$$

4. Normalize the EVSD such that it lies within the range $[0, 1]$

$$\bar{d}_{EVS} = \frac{0.1781}{\sqrt{2 - 2/\sqrt{5}}} = 0.1694 \tag{9}$$

5. Thus the EVSA can be computed as

$$1 - \bar{d}_{EVS} = 1 - 0.1694 = 0.8306 \tag{10}$$

6. And the alignment from G_2 to G_1 can be found as

$$Q^* = {Q_1}^T Q_2 = \begin{bmatrix} 0 & 0 & 1 & 0 & 0 \\ 0 & 0 & 0 & 1 & 0 \\ 0 & 1 & 0 & 0 & 0 \\ 1 & 0 & 0 & 0 & 0 \\ 0 & 0 & 0 & 0 & 1 \end{bmatrix}^T \begin{bmatrix} 1 & 0 & 0 & 0 & 0 \\ 0 & 1 & 0 & 0 & 0 \\ 0 & 0 & 0 & 1 & 0 \\ 0 & 0 & 0 & 0 & 1 \\ 0 & 0 & 1 & 0 & 0 \end{bmatrix} = \begin{bmatrix} 0 & 0 & 0 & 0 & 1 \\ 0 & 0 & 0 & 1 & 0 \\ 1 & 0 & 0 & 0 & 0 \\ 0 & 1 & 0 & 0 & 0 \\ 0 & 0 & 1 & 0 & 0 \end{bmatrix} \tag{11}$$

Figure 2 shows the alignment obtained from above computation.

References

[1] Cha SH. Comprehensive survey on distance/similarity measures between probability density functions. International Journal of Mathematical Models and Methods in Applied Science. 2007;1(2):1.
